# ROS-Dependent Mitochondria Molecular Mechanisms Underlying Antitumor Activity of *Pleurotus abalonus* Acidic Polysaccharides in Human Breast Cancer MCF-7 Cells

**DOI:** 10.1371/journal.pone.0064266

**Published:** 2013-05-14

**Authors:** Xiaolong Shi, Yan Zhao, Yadong Jiao, Tengrui Shi, Xingbin Yang

**Affiliations:** 1 Key Laboratory of Medicinal Resources and Natural Pharmaceutical Chemistry, Ministry of Education, and National Engineering Laboratory for Resource Developing of Endangered Chinese Crude Drugs in Northwest of China, College of Food Engineering and Nutritional Science, Shaanxi Normal University, Xi’an, China; 2 School of Pharmacy, Fourth Military Medical University, Xi’an, China; University of the Witwatersrand, South Africa

## Abstract

**Background:**

A greater reduction in cancer risk associated with mushroom diet rich in fungus polysaccharides is generally accepted. Meanwhile, edible *Pleurotus abalonus* as a member of *Abalone* mushroom family is a popular nutritional supplement that purportedly prevents cancer occurrence. However, these anecdotal claims are supported by limited studies describing tumor-inhibitory responses to the promising polysaccharides, and the molecular mechanisms underlying these properties have not yet been elucidated.

**Methodology/Principal Findings:**

We here fractionated the crude polysaccharide preparation from the fruiting bodies of *P. abalonus* into three fractions, namely PAP-1, PAP-2 and PAP-3, and tested these fractions for antiproliferative activity in human breast cancer MCF-7 cells. The largest PAP-3, an acidic polysaccharide fraction with a molecular mass of 3.68×10^5 ^Da, was the most active in inhibiting MCF-7 cancer cells with an IC_50_ of 193 µg/mL. The changes in cell normal morphology were observed by DAPI staining and the PAP-3-induced apoptosis was confirmed by annexin V/propidium iodide staining. The apoptosis was involved in mitochondria-mediated pathway including the loss of mitochondrial membrane potential (Δψm), the increase of Bax/Bcl-2 ratio, caspase-9/3 activation, and poly(ADP-ribose) polymerase (PARP) degradation, as well as intracellular ROS production. PAP-3 also induced up-regulation of p53, and cell cycle arrest at the S phase. The incubation of MCF-7 cells with antioxidant superoxide dismutase (SOD) and N-acetylcysteine (NAC) significantly attenuated the ROS generation and apoptosis caused by PAP-3, indicating that intracellular ROS plays a pivotal role in cell death.

**Conclusions/Significance:**

These findings suggest that the polysaccharides, especially acidic PAP-3, are very important nutritional ingredients responsible for, at least in part, the anticancer health benefits of *P. abalonus* via ROS-mediated mitochondrial apoptotic pathway. It is a major breakthrough bringing new insight of the potential use of the polysaccharides as health-care food or medicine to provide significant natural defense against human cancer.

## Introduction

Mushroom is a special group of macroscopic fungi with distinctive and visible fruiting body that may grow above or below ground, and many mankind cultures have used mushrooms as a food and medicine since ancient times [1], [2]. In this regard, edible mushrooms have been strongly investigated because naturally occurring wide varieties make up a high proportion in our diet owing to their attractive taste, aroma and nutritional values, and are found to contain large amounts of putative bioactive compounds with their health benefits [3], [4]. Meanwhile, the growing studies have convincingly established the anticancer potential of the promising polysaccharide phytochemicals because the polysaccharides from medicinal fungi or mushrooms provide an important and abundant source of nutraceutical and pharmaceutical compounds due to their notable immunomodulation and antitumor activities, and other medicinal properties [5].

*Pleurotus* species, commonly known as oyster mushrooms, are edible fungi that are cultivated worldwide and have high protein content and gourmet food quality [6]. Meanwhile, edible *Pleurotus abalonus* as a member of *Pleurotus* mushroom family is characterized by its black-headed coremioid imperfect state that is seen on the edge and face of the lamellae, and is a popular nutritional supplement which can reduce cancer risk [7], [8]. Indeed, several recent studies have indicated that the polysaccharides from the mycelia and fruit bodies of different genus *Pleurotus*, *Pleurotus sajor-caju*, *Pleurotus ostreatus*, *Pleurotus citrinopileatus*, and *Pleurotus florida* can inhibit the growth of several types of cancers [7]–[11]. Interestingly, Li et al. also reported that a polysaccharide-peptide complex from the fruiting bodies of *P. abalones* exhibited antioxidant, anti-proliferative and hypoglycaemic activities [12], indicating that *P. abalones* polysaccharides have promising activity for the treatment of cancer. However, so far there is little published information about the antitumor molecular mechanisms of *P. abalonus* polysaccharides on MCF-7 cells [13]. The chemical characteristics of *P. abalonus* polysaccharides are still unclear, and their molecular mechanisms underlying antitumor activity remain poorly understood.

The aim of the present work is therefore to purify the polysaccharide fractions from the fruiting bodies of *Abalone* mushroom (*P. abalonus*) into three main fractions, named PAP-1, PAP-2 and PAP-3, by DEAE-52 ion-exchange column chromatography and subsequently Sephadex G-100 column chromatography, and their antitumor activities in human breast cancer MCF-7 cells were evaluated, and the potential molecular mechanisms were also further studied in vitro. Our results demonstrate that high-molecular mass of PAP-3 is more active in inhibiting the growth of MCF-7 cells, and the effect of the PAP-3 is associated with ROS production leading to mitochondrial dysfunction, ultimately causing cancer cell death. These findings have important implications for the potential use of the promising *P. abalonus* polysaccharides, especially acidic PAP-3, as a therapeutic or prophylactic treatment for human cancer disease.

## Materials and Methods

### Chemicals and Reagents

DEAE-cellulose 52 and Sephadex G-100 were purchased from Whatman Co. (Maidstone, Kent, UK) and Pharmacia Co. (Sweden), respectively. T-series dextrans were purchased from Amersham Pharmacia (Uppsala, Sweden). Dimethyl sulfoxide (DMSO), EDTA, 3-(4,5-Dimethylthiazol-2-yl)-2,5-diphenyltetrazolium bromide (MTT), phenylmethyl-sulfonyl fluoride (PMSF), RNase-A, Tris-HCL, glycine, and propidium iodide (PI) were obtained from Sigma-Aldrich (St. Louis, MO, USA). The primary antibodies against Bax (^#^3331-100), Bcl-2 (^#^3195-100), RPRP (^#^3002-100), and the horseradish peroxidase (HPR)-conjugated goat anti-mouse secondary antibody (^#^6402-05) were obtained from BioVision, Inc. (BioVision, CA, USA) [14]–[16]. The monoclonal antibodies to p53 (^#^2527) , cleaved Caspase-3 (^#^9661), cleaved Caspase-9 (^#^7237), GDPAH (^#^2118), and the horseradish peroxidase (HPR)-conjugated goat anti-rabbit secondary antibody (^#^7074S) were obtained from Cell Signaling Technology, Inc. (Cell Signaling Technology, MA, USA) [17]–[20]. The enhanced chemiluminescence kits were purchased from Pioneer Technology, Inc. (Pioneer Technology, USA). Deionised water was prepared using a Millipore Milli Q-Plus system (Millipore, Bedford, MA, USA). All other chemicals were of the highest grade available.

### Isolation and Purification of *P. abalonus* Polysaccharides

The dried fruiting bodies of *P. abalonus*, an edible mushroom, were purchased from Gutian County, Fujian Province, China and identified according to the identification standard of Pharmacopeia of the People’s Republic of China. Voucher specimens of the materials were deposited at the key laboratory of ministry of education for medicinal resource and natural pharmaceutical chemistry, Shaanxi Normal University, China. The mushroom materials were thoroughly washed with tap water, air-dried, and finely powdered. The crude polysaccharides were isolated as previously described [21]. Briefly, the dried powder (250 g) was defatted with anhydrous ethanol. After the mixture was filtered, the residues were dried in air and then were extracted with hot water (1∶10, w/v) at 80°C for three times, 1 h each time. The combined extracts were pooled, concentrated to 30% of the original volume under reduced pressure and then centrifuged at 2000 rpm for 15 min. The supernatant was collected and three volumes of 95% alcohol were added slowly by stirring to precipitate the polysaccharides, and then kept at 4°C overnight. Finally polysaccharide pellets were obtained by centrifugation at 4000 rpm for 15 min and repeatedly washed sequentially with anhydrous ethanol, acetone and diethyl ether. The refined polysaccharide pellets were completely dissolved in an appropriate volume of distilled water and intensively dialysed for 2 days against distilled water (MW>8000 Da). The retentate portion was deproteinised by a freeze-thaw process (FD-1, Henan Yuhua Instrument Co., China), which was repeated eight times, followed by filtration. Finally, the filtrate was lyophilised to yield the crude polysaccharides (PAP).

PAP (200 mg) was dissolved in distilled water, and then filtered through 0.45 µm membrane. The crude polysaccharide solution was subjected to a DEAE-52 cellulose column (2.6 cm×50 cm) with a stepwise elution of NaCl solution (0, 0.05, 0.1, 0.3, and 0.5 M). Fractions were collected, and monitored with the phenol-sulfuric acid method. The three main fractions (P-1, P-2 and P-3) eluted with 0, 0.05 and 0.1 M NaCl were collected, dialyzed, and lyophilized, respectively, and were further purified with a Sephadex G-100 column (2.6 cm×60 cm), and the elution was performed with distilled water at a flow rate of 0.5 mL/min. Finally, PAP-1, PAP-2 and PAP-3 were obtained from P-1, P-2 and P-3, respectively. The fractions obtained were combined according to the total carbohydrate content quantified by the phenol-sulfuric acid method.

### Characterization of Polysaccharide Fractions

#### Ultraviolet analysis

PAP-3 was dissolved in distilled water with magnetic stirring until complete solubilization. UV spectroscopy was recorded using a UV-2450 spectrophotometer (Shimadzu, Japan) in the range 200–400 nm.

#### Molecular weights of polysaccharides

The molecular weight of PAP-3 were determined by high performance size exclusion chromatography (HPSEC) using a Shimadzu LC-2010A HPLC system equipped with a size exclusion chromatography (SEC) column (Shodex SB-804 HQ, Showa Denko, Kawasaki, Japan). PAP-3 (2 mg/mL, 20 µl) was injected into the column and eluted by ultrapure water at a ﬂow rate of 0.8 mL/min. The molecular weight of PAP-3 was estimated by the comparison to a calibration curve prepared with the T-series Dextran standards (Dextran T-10, T-40, T-70, T-90, T-100, and T-200).

#### Monosaccharides composition

The monosaccharides of PAP-3 were analyzed by HPLC as previously described [21]. The analysis of monosaccharides was performed on a Shimadzu LC-2010A HPLC system. The analytical column used was a RP-C_18_ column (4.6 mm i.d.×250 mm, 5 µm, Venusil, USA).

### Cell Lines and Cell Culture

Human breast carcinoma cell MCF-7 line and normal mammary epithelial cell H184B5F5/M10 cell line were obtained from Cell Bank of Institute of Biochemistry and Cell Biology, Chinese Academy of Sciences (Shanghai, China). The cells were cultured in DMEM medium supplemented with 10% FBS, 10 µg/mL streptomycin and 100 U/mL penicillin and maintained at 37°C with 5% CO_2_ in a humidified atmosphere.

### Anti-proliferation Studies of Polysaccharide Fractions

#### MTT assay

Human breast carcinoma MCF-7 cells and H184B5F5/M10 cells were plated at density of 5×10^3^ cells/well in 96-well plates, and after 24 h of incubation, PAP-1, PAP-2, and PAP-3 at a series of concentrations (0, 25, 50, 100, 200, and 400 mg/mL) were added to the wells and incubated for 48 h [22]. After the exposure period, 10 µL of MTT (5 mg/mL) in PBS solution was added to each well at a final concentration of 0.5 mg/mL and then the plate was further incubated for 4 h. MTT-containing media were removed, and 150 µL of solution containing 10% SDS plus 0.01 M HCl and 5% isobutyl alcohol was added to each well and mixed thoroughly to dissolve the formed crystal formazan. After incubation overnight at 37°C to ensure that all crystals were dissolved, the light absorption was measured at 570 nm using an enzyme-linked immunosorbent assay reader (Rayto-RT6000, Guangdong, China). Viability was expressed as a percentage of absorbance values in treated cells to that in control cells.

#### Lactate dehydrogenase (LDH) assay

The leakage into the media of LDH, as an indicator of cell membrane injury [22], [23], was detected with an assay kit (Jiancheng BioEngineering, Nanjing, China) according to the manufacturer instructions. Briefly, at the end of the incubation 20 µL of culture supernatants by different treatment was taken out for the activity analysis of extracellular LDH. Each sample was detected and the absorbance was read at wavelength 450 nm and the results were also expressed as the percentage of LDH leakage from treated cells versus control cells.

### Morphological Study

Cells were washed with PBS and fixed in 4% paraformaldehyde for 30 min at room temperature and then washed again with PBS. The fixed cells were incubated with 1 µg/mL DAPI solution for 20 min at room temperature in the dark. Stained solution was washed out, and the cells were visualized with a fluorescence microscope (Leica DMIL LED, Leica, Germany) for determination of nuclear morphological changes [24].

### Cell Cycle Analysis

Control and treated cells (1×10^6^) were collected by centrifugation at 400 g for 5 min and washed with PBS, and the cells were fixed and permeabilized by addition of ice-cold 70% (v/v) ethanol and kept overnight at 4°C. The fixed cells were washed with PBS and suspended in 1 mL PBS. Cells were analyzed after incubation with RNase A (50 µg/mL) and PI (40 µg/mL) for 30 min at 37°C in the dark. The PI fluorescence was measured using flow cytometry (FACSCalibur, Becton Dicknson, USA) [25].

### Apoptosis Assay

Viable cells along with the cells in the early and late phases of apoptosis and necrosis were quantitated using Annexin V-FITC/PI Apoptosis Detection Kit (BestBio, Shanghai, China) as described by the manufacturer’s instruction. Briefly, cells (1×10^6^) were collected and washed twice with PBS and suspended in 400 µl of binding buffer (containing 5 µl of annexin V-FITC and 10 µl of PI). Thereafter, the samples were incubated in the dark for 10 min at 4°C, and then analyzed on a Guava EasyCyte Plus Flow Cytometry System (Millipore).

### Detection of Mitochondrial Membrane Potential (ΔΨm)

The mitochondrial membrane potential (ΔΨm) was measured by ﬂow cytometer using the cationic lipophilic green ﬂuorochrome Rhodamine 123 [23]. Cells were harvested, washed twice with PBS, incubated with 1 µM Rhodamine 123 at 37°C for 30 min, and washed twice with PBS. Fluorescence was determined by a Guava EasyCyte Plus Flow Cytometry System.

### Measurement of ROS

Intracellular production of reactive oxygen species (ROS), namely hydrogen peroxide (H_2_O_2_) and superoxide anion (O_2_^•−^), were measured using DCF-DA and DHE probes, respectively [22], [26]. After incubation with PAP-3 for 6 h, 12 h and 24 h, cells were stained with DCF-DA or DHE at the final concentration of 5 µM, and reacted at 37°C for 30 min, respectively. ROS production of MCF-7 cells was subjected to evaluate by both a laser scanning confocal microscope (Leica TCS-SP5, Heidelberg, Germany) and a Guava EasyCyte Plus Flow Cytometry System.

### Western Blot

MCF-7 cells (5×10^5^) were plated in a 25 cm^2^ cell culture flask. After 24 h, the cells were treated with PBS vehicle alone or PAP-3 for indicated time, and then the cells were collected and lysed with 0.1 mL of cold lysis buffer (150 mM NaCl, 50 mM of pH 7.4 Tris, 1 mM EDTA, 1% Triton X-100, 0.5% SDS, 0.01% PMSF). The cell lysate was centrifuged at 12 000 g for 30 min at 4°C. Protein concentrations were determined with a BCA protein assay kit (BestBio, Shanghai, China) using bovine serum albumin as the standard. The proteins were separated by SDS-PAGE and transferred to a polyvinylidene difluoride (PVDF) membrane. After nonspecific binding sites were blocked with 5% nonfat dry milk in PBS for 60 min, the transferred membrane was incubated at 4°C overnight with primary antibodies [1∶500]. The membrane was extensively washed by PBST (0.1% Tween-20 in PBS buffer), followed by incubation with horseradish peroxidase-conjugated goat anti-mouse or -rabbit secondary antibody [1∶5000] for 1 h. Proteins of interest were incubated with ECL detection reagent and developed by exposure to X-ray films. The protein expression was quantified densitometrically using ImageJ software (version 1.43, National Institutes of Health, USA).

### Statistical Analysis

All the determinations were performed in triplicate, and data were expressed as means ± SD. IC_50_ values were calculated by regression analysis. The data were subjected to an analysis of variance (ANOVA, *p*<0.05), and Duncan’s multiple range tests (SPSS, version 13.0). A significant difference was judged to exist at a level of *p*<0.05.

## Results

### Preparation of PAP Fractions and their Cytotoxic Effects on MCF-7 Cells

**Figure 1:**
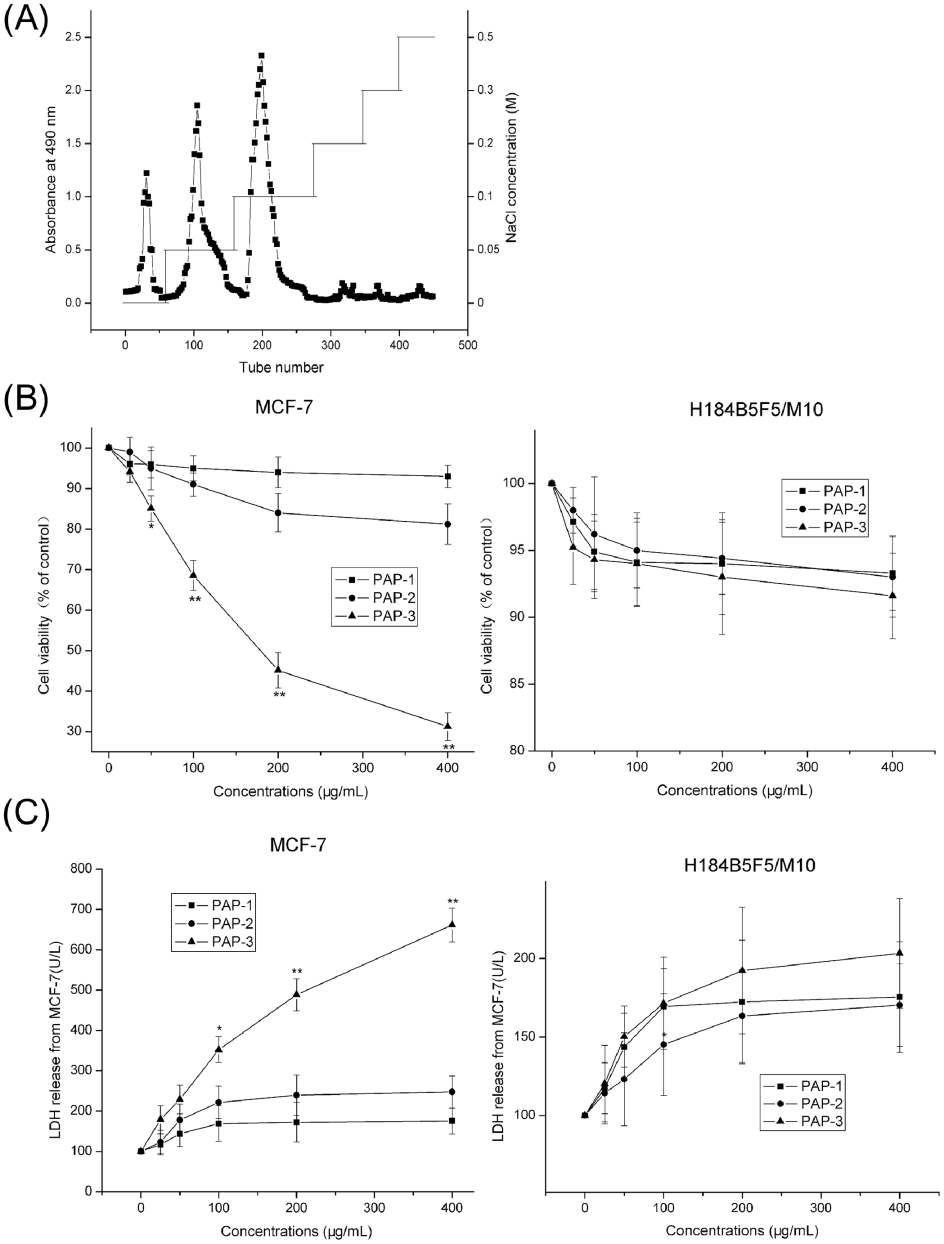
Chromatographic fractionation of polysaccharides (A), and cytotoxicity of three fractions (B) and LDH analysis (C). Crude water extract of *P. abalonus* was prepared and separated on DEAE-52 chromatographic column with gradient of NaCl solution. The cytotoxicity of PAP-1, PAP-2 and PAP-3 on MCF-7 and H184B5F5/M10 cells was assayed by MTT assay, and LDH leakage analysis was performed as described in Materials and Methods. Each bar represents the mean ± SD of three independent experiments. *p<0.05 and **p<0.01 indicate statistically significant differences versus control group.

The water-soluble crude polysaccharides were extracted from the fruiting bodies of *P. abalonus* by hot water extraction and ethanol precipitation. The extracts were further purified through with DEAE-52 cellulose column based on the difference of ionic groups present in *P. abalonus* polysaccharide molecules, and three major fractions eluted with 0, 0.05, 0.1 M sodium chloride solutions were collected, respectively (**Figure 1A**). The fractionated elution was subjected to a further purification by gel-filtration chromatography on Sephadex G-100 column, and then concentrated, dialyzed and lyophilized to afford PAP-1, PAP-2 and PAP-3 for further bioactivity assay, respectively. PAP-1 eluted with deionized water was known as the neutral polysaccharides, whereas PAP-2 and PAP-3 eluted with 0.05 M and 0.1 M NaCl were known as acidic polysaccharides [27].

A comparative study on the viability of human breast carcinoma MCF-7 cells treated with various concentrations of PAP-1, PAP-2 and PAP-3 for 48 h was performed and the results were given in **Figure 1B** as observed by MTT assay. As revealed by the growth curves, PAP-1 and PAP-2 at all the tested concentrations of 0–400 µg/mL did not exhibit prominent anti-proliferation effect on MCF-7 cells, as compared to the untreated cells (*p*>0.05), while PAP-3 revealed a dose-dependent efficiency in lowering the survival rates of MCF-7 cells with an IC_50_ of 193 µg/mL for 48 h (*p*<0.01). However, anti-proliferation effect of PAP-1, PAP-2 and PAP-3 on H184B5F5/M10 normal mammary epithelial cells was not observed at the same tested concentrations for 48 h (**Figure 1B**). To further confirm the proliferation inhibitory effects of PAP-1, PAP-2 and PAP-3 on MCF-7 cells, LDH assay was also performed as another indicator of MCF-7 and H184B5F5/M10 cytotoxicity. As shown in **Figure 1C**, LDH leakage of MCF-7 cells was significantly increased with the presence of PAP-3 (*p*<0.01), while PAP-1 and PAP-2 have little impact on it (*p*>0.05), and this cytotoxic effect was not observed in normal H184B5F5/M10 cells. The results imply that PAP-3 is the only one with antiproliferative effects, which is consistent with the MTT results. In light of this finding, PAP-3 with high sensitivity to MCF-7 cancer cells may be amenable to be tested in its chemical characterization and the molecular mechanism of anticancer effects.

### Chemical Characterization of PAP-3

**Figure 2:**
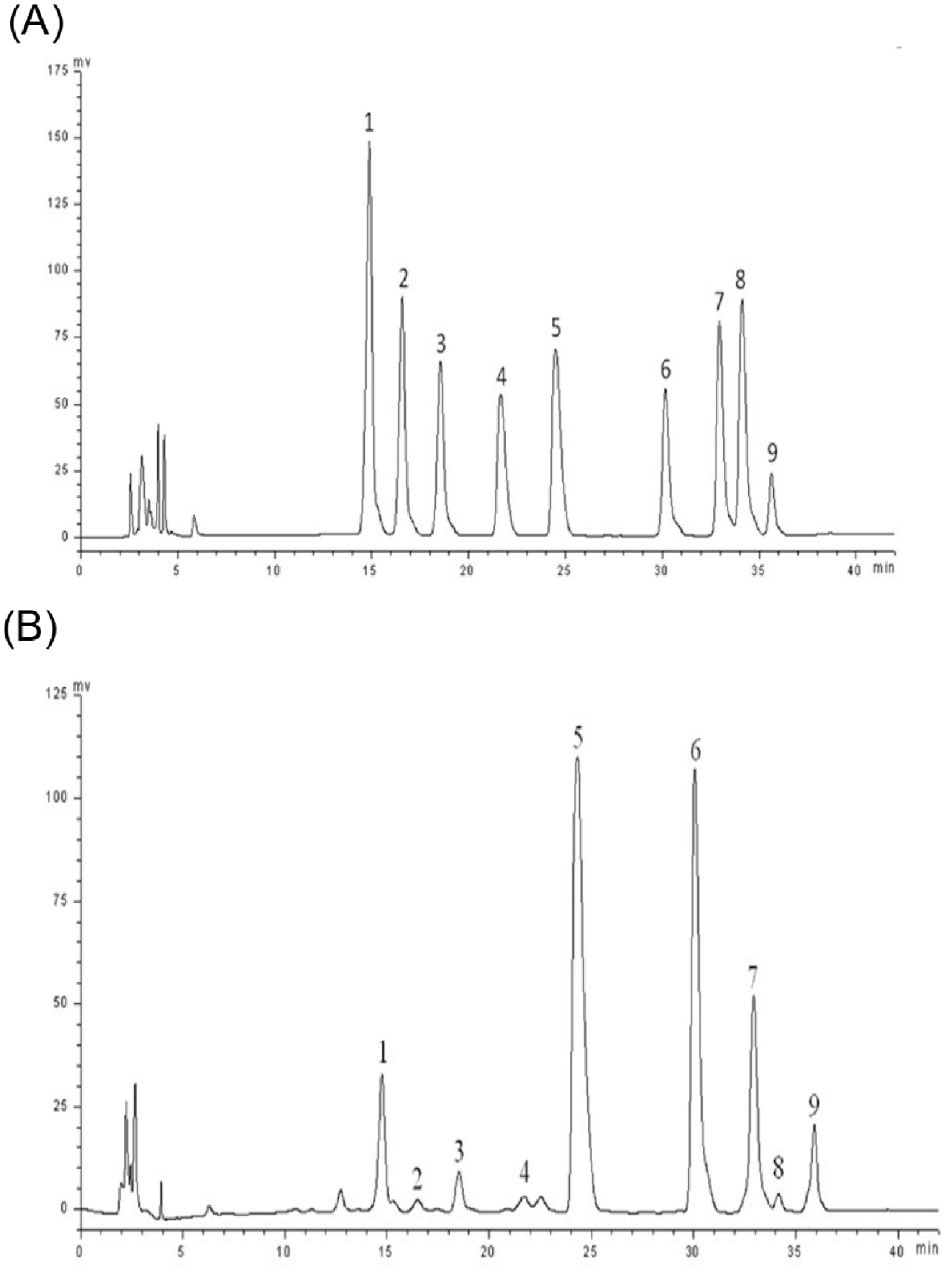
The HPLC chromatograms of standard monosaccharides (A) and component monosaccharides released from PAP-3 (B). PAP-3 was hydrolyzed into component monosaccharides with trifluoroacetic acid at 100°C for 8 h and subsequently was labeled with 1-phenyl-3-methyl-5-pyrazolone (PMP), and then the PMP-labeled monosaccharide were separated and identified by HPLC-UV at 250 nm as described in the experimental section. Peaks: 1. mannose, 2. ribose, 3. rhamnose, 4. glucuronic acid, 5. galacturonic acid, 6. glucose, 7. galactose, 8. arabinose, 9. fucose (internal standard).

PAP-3 has no absorption at 280 nm and 260 nm in the UV spectrum (data not shown), indicating the absence of protein and nucleic acid. The average molecular weight value of PAP-3 was estimated to be 3.68×10^5 ^Da by HPSEC. Furthermore, the HPLC analysis of monosaccharide composition of PAP-3 was shown in **Figure 2**, and PAP-3 was composed of mannose, ribose, rhamnose, glucuronic acid, galacturonic acid, glucose, galactose, and arabinose, and their corresponding mole percentages were 7.8%, 1%, 2.7%, 1.3%, 41.7%, 29.8%, 14.6% and 1.1% of all the quantitative monosaccharides, respectively. This monosaccharide compositional analysis clearly implied that PAP-3 was mainly composed of galacturonic acid, up to 41.7% in the molar percentage of total compositional carbohydrates, followed by glucose (29.8%) and galactose (14.6%), accounting for up to 86.1%. As a result, PAP-3 was for the first time identified as the typically acidic heteropolysaccharide fraction.

### Apoptotic Induction on MCF-7 cells by PAP-3

**Figure 3:**
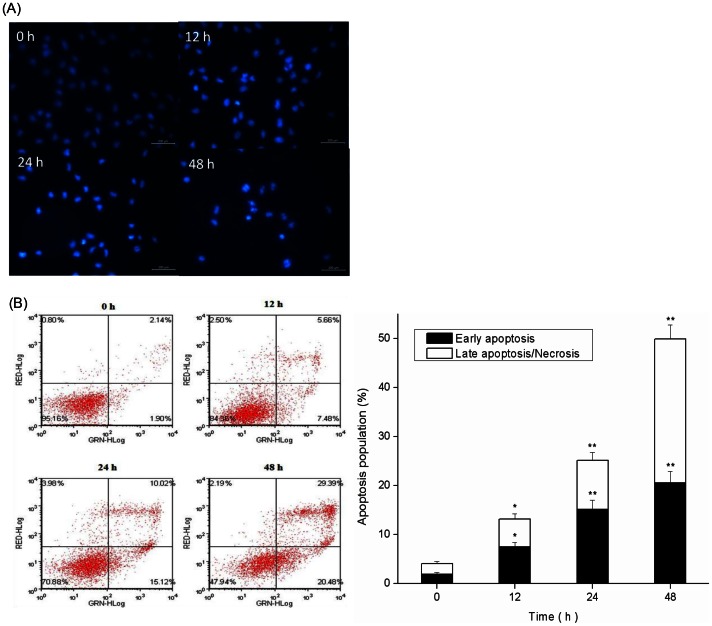
PAP-3 induced apoptosis on MCF-7 cells. (A) MCF-7 cells were treated with 200 µg/mL PAP-3 for 12, 24, and 48 h, and the morphological changes were determined by ﬂuorescence microscopy. (B) Cells were double-stained with annexin V-FITC and PI, and then cells were analyzed by ﬂow cytometry. All experiments were done independently in triplicate per experimental point, and representative results are shown. The results represent the mean ± SD of three independent experiments. *p<0.05 and **p<0.01 indicate statistically significant differences versus control group.

To investigate whether the proliferative inhibition or cell death was caused by apoptosis, an assay for morphological feature was performed. In many cell types, apoptosis is characterized by a specific sequence of nuclear changes culminating in chromatin condensation and nuclear fragmentation [28]. As shown in **Figure 3A**, DAPI staining showed that the nucleus of untreated control cells were large and round without condensation or fragmentation, whereas the nucleus from the PAP-3 treated cells were condensed and fragmented, as is typical in apoptosis. The extent of the changes in cell morphology and density depended on the treated time of PAP-3 (**Figure 3A**). Furthermore, the apoptosis induction ability of PAP-3 was further confirmed and quantified by FACS analysis after staining with Annexin V/PI (**Figure 3B**). As early as 12 h, the exposure to 200 µg/mL PAP-3 resulted in about 7.5% of the cells going into early apoptotic phase (*p*<0.05). With the increase in the exposure duration up to 24 and 48 h, an increase in both early apoptotic cells and late apoptotic cells was observed in PAP-3 treated cells, respectively (*p*<0.01). Taken together, these results suggest that PAP-3 does trigger apoptotic cell death in MCF-7 cells.

### Effects of PAP-3 on Mitochondria-mediated Apoptosis Pathways

**Figure 4:**
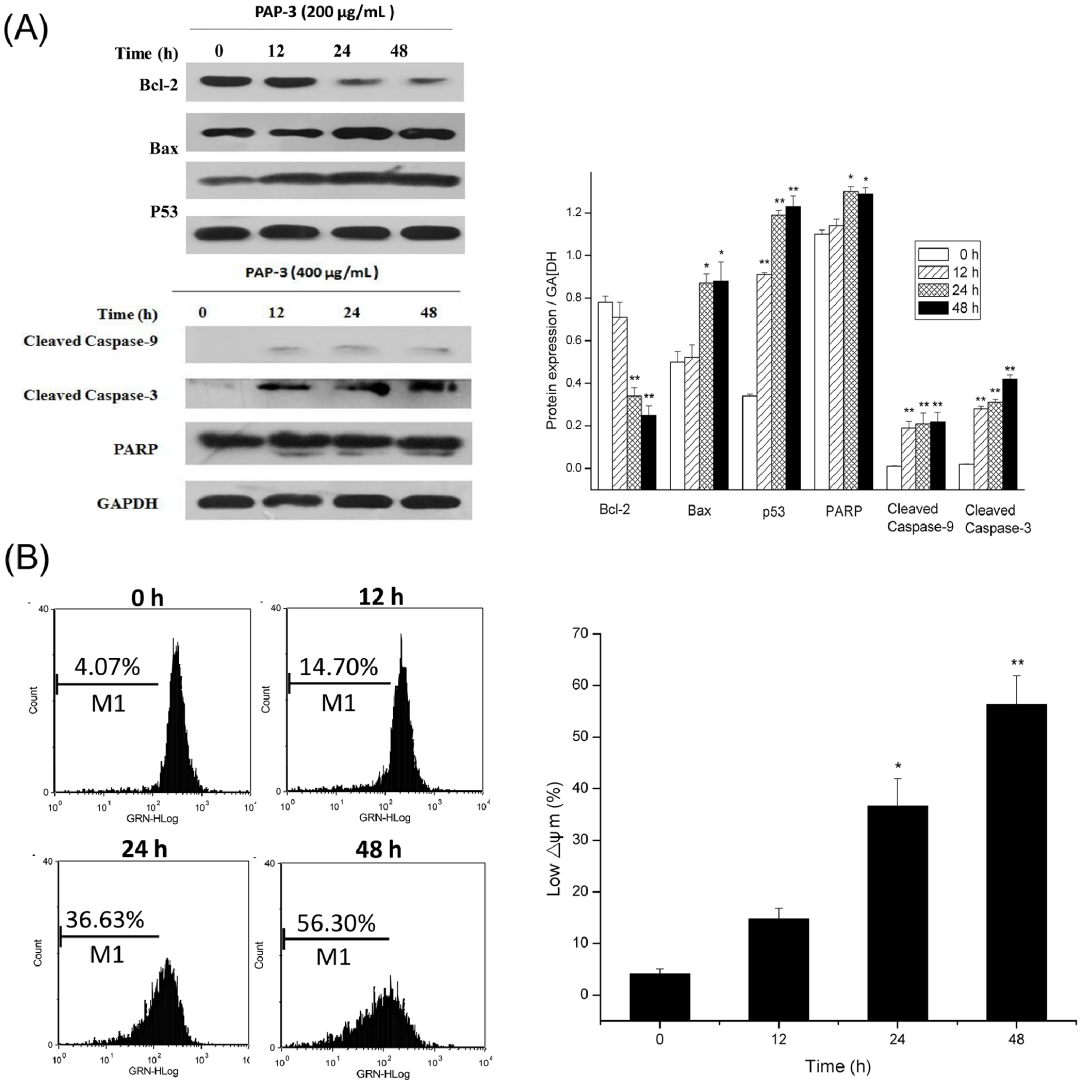
PAP-3 induced mitochondrial apoptotic pathway. (A) The protein expression was determined with or without 200 µg/mL PAP-3 by Western blot, with GAPDH as loading control. (B) Evaluation of mitochondria membrane potential in MCF-7 cells was done with Rhodamine 123 stain by ﬂow cytometry. The percentage of M1 reflects the reduction of ΔΨm. All experiments were done independently in triplicate per experimental point, and representative results are shown.

Apoptosis mediated by mitochondria is the best known intrinsic apoptosis pathway [29]–[32]. To determine whether the mitochondrial apoptotic signaling pathway was involved in PAP-3 induced apoptosis, expression patterns of pro-apoptotic and anti-apoptotic Bcl-2 family proteins were investigated. As shown in **Figure 4A**, the exposure of MCF-7 cells to 200 µg/mL PAP-3 up-regulated the expression of pro-apoptotic protein Bax, whereas the expression of anti-apoptotic protein Bcl-2 was down-regulated with the increasing time of PAP-3 treatment. The imbalance of Bcl-2 and Bax protein expression could be influenced by proteins of p53, which is one of the major mechanisms underlying the ultimate fate of cells with respect to apoptosis [33], [34]. Here, western blot analysis revealed that the p53 protein level was elevated after PAP-3 treatment in MCF-7 cells (**Figure 4A**). Moreover, the cleaved caspase-9 was firstly observed at 12 h, which was accompanied by the activation of caspase-3 and PARP after the treatment of PAP-3. It is also well known that the decrease of mitochondrial membrane potential (ΔΨm) is an important step in the induction of apoptosis by this mechanism [35]. As shown in **Figure 4B**, when MCF-7 cells were treated with 200 µg/mL PAP-3 for indicated periods, the population that lost ΔΨm was from 4.1% to 14.7% at 6 h (*p*<0.05), and up to 56.3% at 24 h (*p*<0.01), indicating the collapse of ΔΨm. These observations strongly indicate that PAP-3 exerts pro-apoptotic effects through a mitochondrial-mediated apoptosis pathway.

### Effects of PAP-3 on Cell Cycle Arrest

**Figure 5:**
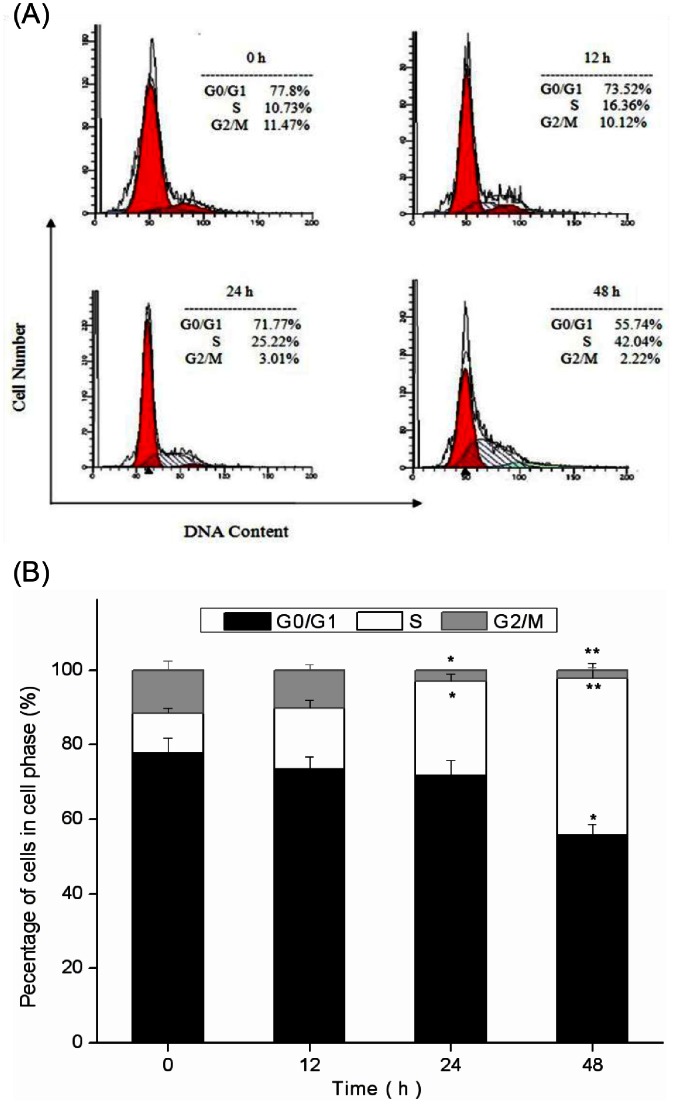
Cell cycle analysis of PAP-3-treated cells. Cells were harvested and fixed in 70% alcohol and then stained with propidium iodide. Finally the stained cells were analyzed using a flow cytometer.

Besides apoptosis, inhibition of cell cycle progression is another important strategy to controlling cancer cell growth [34]. As shown in **Figure 5**, PAP-3 treatment induced a strong S-phase arrest in a time-dependent manner (*p*<0.01). When MCF-7 cells were incubated with 200 µg/mL PAP-3 for 0, 12, 24, and 48 h, the relative percentages of cells staying at the S phase were 5.7%, 16.4%, 25.2%, and 42%, respectively. This increase in the S-phase cell population was accompanied by a concomitant decrease in the G0/G1 and G2/M phase cell populations. These results indicated that PAP-3 caused cell cycle arrest at the S phase.

### The Role of ROS on PAP-3 Induced Apoptosis

**Figure 6:**
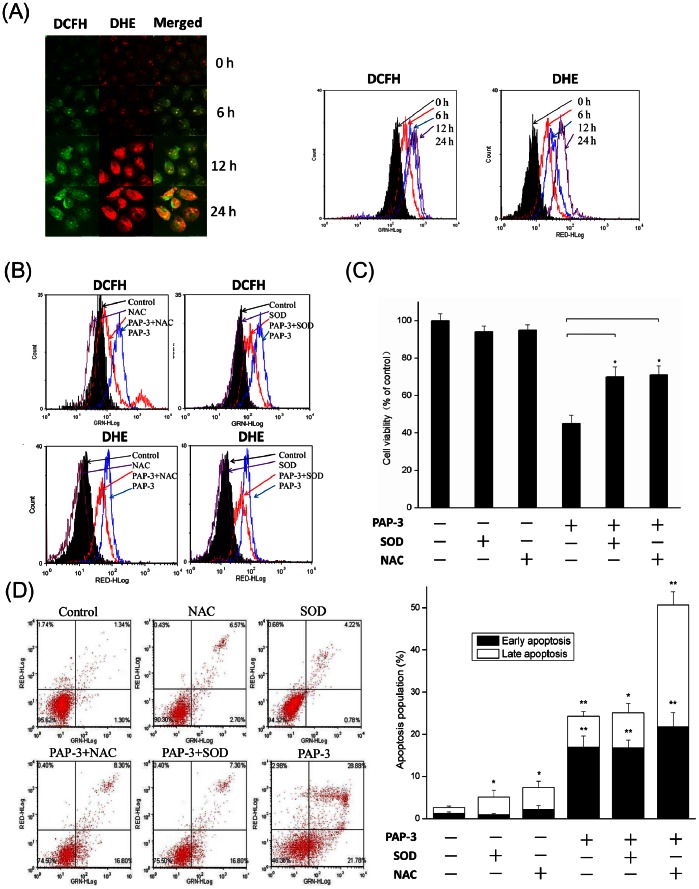
PAP-3 increased ROS production in MCF-7 cells. (A) Cells were treated with or without 200 µg/mL PAP-3 for 0, 6, 12, and 24 h, and the ROS levels were measured by both the Laser Scanning Confocal Microscopy system and ﬂow cytometry after an incubation with DCF-DA (for H_2_O_2_) or DHE (for O_2_^•−^) ﬂuorescence probe stain. (B) SOD and NAC eliminated PAP-3-induced ROS generation. Cells were protected by SOD (30 U/mL), and NAC (1 mM) with 1 h pretreatment when co-incubated with 200 µg/mL PAP-3 for another 24 h, and the ROS levels were stained with DCF-DA or DHE. (C) Impact of SOD (30 U/mL) and NAC (1 mM) on the viable cell was determined by trypan blue dye exclusion assay. Cells were pretreatment with SOD (30 U/mL), and NAC (1 mM) for 1 h, then co-treated with 200 µg/mL PAP-3 for another 48 h. (D) Impact of SOD and NAC on the apoptotic value was determined by Annexin V-FITC/PI staining. All experiments were done independently in triplicate per experimental point, and representative results are shown.

The generation of ROS in mitochondria has been reported to be implicated in the induction of apoptosis by several phytochemicals [36]–[38]. Herein, we tested whether the apoptosis caused by PAP-3 was associated with the change in intracellular ROS levels. To examine this hypothesis, cells were loaded with the fluorescent probes DCF-DA and DHE, which can detect H_2_O_2_ and O_2_^•−^, respectively, and assessed by both Laser Scanning Confocal Microscope and flow cytometry in the absence or presence of 200 µg/mL PAP-3 for 6, 12 and 24 h. Compared with the control group, the generation of intracellular H_2_O_2_ and O_2_^•−^ significantly increased after exposure to PAP-3 (**Figure 6A**).

To further validate that ROS was involved in PAP-3 induced apoptotic pathway of MCF-7 cells, enzymatic and nonenzymatic ROS scavengers were pretreated with MCF-7 cells. As expected, the presence of the 30 U/mL enzymic scavenger SOD or 1 mM antioxidant NAC abolished the accumulation of intracellular ROS caused by PAP-3 (**Figure 6B**). As shown in **Figure 6C**, the antiproliferative effect of PAP-3 was largely reversed by pretreatment with SOD or NAC. The viable cells was about 71% and 74% by pretreatment with SOD or NAC, respectively, compare with only about 45% by pretreatment with PAP-3 alone (*p*<0.05). Annexin V-FITC/PI double staining assay also revealed that the pretreatment with SOD or NAC could protect MCF-7 cell from PAP-3 induced apoptosis (**Figure 6D**). These results indicate that PAP-3-induced apoptosis is involved in the increase in intracellular ROS oxidative stress of MCF-7 cells.

## Discussion

Medicinal or edible mushroom consumption is associated with the improvement of human health, especially for the cancer prevention [39]–[41]. Interestingly, the active constituents that are involved in these effects of mushrooms are considered to be polysaccharides, which can induce tumor cell death in several cancer types [13], [39]. In the present study, using ion-exchange and subsequent gel filtration chromatography the parent *P. abalonus* polysaccharides from the fruiting bodies of *P. abalonus* were separated into three main fractions, named PAP-1, PAP-2 and PAP-3. To compare the biological activities of the fractions, we evaluated anti-tumor effects. Interestingly, in this study only one of the isolated fractions, namely high-molecular weight of PAP-3, could remarkably suppress the growth of human breast carcinoma MCF-7 cells, as shown in **Figure 1**, suggesting that it is highly feasible to improve biological activity of *P. abalonus* polysaccharides by the separation of more active fractions and even chemical manipulation. Traditionally, the ingestion of edible *P. abalonus* is associated with the cancer prevention, and *P. abalones* polysaccharides have also been shown to exhibit antitumor effects [7], [8], [12], [13]. Herein, PAP-3 was firstly shown to be one of the main active ingredients responsible for the anticancer effect of *P. abalonus*, and represent promising candidates as a therapeutic or prophylactic treatment for cancer.

It is widely recognized that the molecular weight and monosaccharide composition are considered as two important factors related to the anti-cancer activities of natural polysaccharides. In this regard, a recent study has documented the ability of the polysaccharides with relatively high molecular weight exhibited high inhibitory activity against S-180 tumor cells [42]. However, whether natural polysaccharides with large-molecular mass can be absorbed into the bloodstream to exert the putative anticancer effects is still not clear. In fact, a few mushroom polysaccharides have been taken to clinical assessment in humans [43]. In 1985, lentinan with a high molecular weight of 5×10^5^ Da was approved and produced as an adjuvant for the treatment of cancer in Japan [43]. It has also been suggested that mushroom polysaccharides with high anti-tumor activities are mostly heteropolysaccharides [44]. Our results were in good agreement with the above reports, the highest molecular weight of PAP-3 with 3.68×10^5 ^Da, as acidic heteropolysaccharide, was shown to have the highest antitumor activity in *P. abalonus* polysaccharide fractions. Interestingly, PAP-3 was shown to be composed primarily of galacturonic acid and glucose, which accounted for 41.7% and 29.8% of all the quantitative monosaccharides, respectively. These structural features may be responsible for the high anti-proliferative activity of PAP-3 in MCF-7 cells. This high sensitivity of acidic PAP-3 to MCF-7 cancer cells also makes it a promising heteropolysaccharide fraction for the development of novel effective cancer preventive or therapeutic agents.

Another interesting feature of natural polysaccharides is represented by their pro-apoptotic activities, and represents a promising strategy for controlling several malignancies [29], [32]. Apoptosis is a process of programmed cell death, which is characterized by various biochemical and morphological changes, including cell shrinkage, chromatin condensation, internucleosomal DNA fragmentation, and formation of “apoptotic bodies” [28]. To evaluate the effect of PAP-3 on the nuclear morphology, DAPI staining was performed. As shown in **Figure 3A**, the nuclei of cells treated with 200 µg/mL PAP-3 was darkly stained, and thus fluoresced brightly, indicating the condensation of chromatin. Furthermore, the apoptosis induction ability of PAP-3 was confirmed and quantified by FACS analysis after staining with Annexin V-FITC/PI. A cell population with annexin V positive and PI negative is considered as an early apoptotic population, whereas a cell population with both annexin V and PI positive is considered as a late apoptotic/necrotic population. As depicted in **Figure 3B**, the late apoptotic cells were concomitantly increased with the increased time of PAP-3 treatment.

Mitochondria are thought to be the major pathway for apoptosis, and therefore, targeting the mitochondria is a novel strategy for cancer therapy [24], [31]. Mitochondrial mediate-apoptosis is highly regulated by the Bcl-2 family proteins comprising both anti-apoptotic (Bcl-2, Bcl-XL) and proapoptotic members (Bax, Bak), and the balance between the expression levels of pro- and anti-apoptotic proteins is critical for cell survival or cell death [23], [45]. In our hands, PAP-3 treatment resulted in a significant increase in Bax expression, and a decrease in Bcl-2 expression, suggesting that the change in the ratio of pro-apoptotic and anti-apoptotic Bcl-2 family proteins might contribute to the mitochondria-mediate apoptosis. It is widely recognized that the Bcl-2 family of proteins play a critical role in switching the balance between survival and death largely by regulating mitochondrial membrane permeability, and the major consequences of this change of permeability are the loss of the mitochondrial transmembrane potential (ΔΨm) [35], [46]. As investigated by FCS using Rhodamine 123 staining, as early as 12 h there was a decrease in Rhodamine 123 ﬂuorescence after treatment with PAP-3, compared with the untreated control, indicating that PAP-3 induced ΔΨm disruption in MCF-7 cells. In addition to the loss of ΔΨm, the change of permeability can lead to the release of apoptosis factors such as cytochrome c, which triggers the activation of caspase-9, followed by activation of effector caspase-3 and subsequently the cleavage of PARP and final induction of apoptosis [37], [46]. Our western blot analysis also showed that aspase-9/3 and PARP were all involved in PAP-3 induced apoptosis in MCF-7 cell. These results clearly indicate that PAP-3 induces apoptosis via mitochondrial pathways.

p53, also known as the “guardian of the genome”, has been shown to play a critical role in intrinsic tumor suppression via cell cycle arrest and induction of apoptosis [24], [30], [33], [34]. For cell cycle arrest, p53 exerts its effects through transcriptional activation of target genes such as the cyclin-dependent kinase (CDK) inhibitor p21, and then lead to cell cycle arrest [24], [34]. In addition, it was also found that there was an increase of p53 expression in PAP-3 treated cells by western blot assay. Conformably, cell cycle analysis also showed PAP-3 caused cell cycle arrest at the S phase, suggesting that PAP-3 blocked proliferation of MCF-7 cells by arresting the cells in the S phase of the cell cycle, which might be regulated by p53. Besides cell cycle arrest, p53 can directly stimulate mitochondrial perturbations. The Bcl-2 family has been shown to be a p53 target, and Bax is up-regulated and Bcl-2 is down-regulated in a number of systems during p53-mediated apoptosis [30]. In the present study, an increase in Bax and p53 expression, and a decrease in Bcl-2 expression were all observed in the MCF-7 cells treated with PAP-3, indicating that p53 is involved in the apoptotic effect of PAP-3.

In mammalian cells, increased cellular ROS production has been suggested to be responsible for the depolarization of mitochondrial ΔΨm and subsequent cell death [46], [47]. O_2_^•−^, H_2_O_2_, hydroxyl radical (HO^•^) and hypochlorous acid are included among ROS that is natural by-products of normal cell metabolism [36], [38], [46]. Evidence is accumulating which indicates that many chemotherapeutic agents may be selectively toxic to tumor cells because they increase oxidant stress and enhance these already stressed cells beyond their limit [37], [38], [46], [47]. Previous studies indicate that the production of ROS is upstream factors for regulating apoptosis. To investigate if the mitochondrial dysfunction observed in MCF-7 cells treated with PAP-3 is promoted by ROS production, we measured ROS levels using the cell-permeable dye DCF-DA and DHE. The results showed that the apoptotic effect of PAP-3 on MCF-7 cells was associated with an early elevated level of intracellular ROS in a time- or concentration-response manner. As shown in Figure 6A, the levels of H_2_O_2_ and O_2_^•−^ in 200 µg/mL PAP-treated cells for 24 h were elevated by 5.4-fold and 5-fold, compared to the untreated control cells, respectively. Moreover, to further confirm the finding that the apoptotic effect of PAP-3 was mediated by ROS, antioxidants NAC and SOD were also employed. Both NAC and SOD inhibited the accumulation of intracellular ROS induced by PAP-3, and both could ensure cell survival as shown by trypan blue exclusion and significantly reduced cell apoptosis as demonstrated by Annexin V-FITC/PI staining. These results indicate that PAP-3-induced apoptosis is associated with ROS generation. However, the protection of SOD and NAC was incomplete, as reflected by the fact that the pretreatment with SOD or NAC still existed about 30% cell death, suggesting that both ROS-dependent and -independent mechanism may be involved in PAP-3 induced apoptosis, and ROS-independent mechanism needs to be further investigated in the future.

In conclusion, this is the first study to show that PAP-3, an acidic polysaccharide fraction derived from the fruiting bodies of *P. abalonus*, exhibit anti-proliferation of human breast cancer MCF-7 cells via cell cycle arrest at the S phase and cellular apoptosis mediated by intracellular ROS-dependent mitochondrial pathways. The observed antitumor effect indicates the possibility that PAP-3 may provide an effective and alternative treatment strategy for cancer prevention. Understanding the involvement of the active polysaccharide fraction in human cancer treatment is an attractive challenge and yet requires further investigation.
